# Unexpected gastric perforation during endoscopic submucosal tunnel dissection for early circumferential esophageal cancer

**DOI:** 10.1055/a-2106-1012

**Published:** 2023-06-22

**Authors:** Jingjing Lian, Aiping Xu, Tao Chen, Haibin Zhang, Meidong Xu

**Affiliations:** Endoscopy Center, Department of Gastroenterology, Shanghai East Hospital, Tongji University School of Medicine, Shanghai, China

Endoscopic submucosal dissection (ESD) has been widely accepted as an effective and minimally invasive treatment for superficial esophageal neoplasms. Mallory–Weiss tear (MWT) during esophageal ESD has been recognized as a possible complication, with an approximate incidence of 5.4 % according to Chen et al. 1 Here, we report a very rare case in which Mallory–Weiss tear led to severe gastric perforation during esophageal ESD.

An 82-year-old man was admitted for treatment of a circumferential superficial esophageal neoplasm ( Fig. 1 ). Endoscopic submucosal tunnel dissection (ESTD) – a modified ESD method – was performed by the highly skilled endoscopist, under general anesthesia. Carbon dioxide was used for insufflation. The procedure has been described previously 2 .

**Fig. 1:**
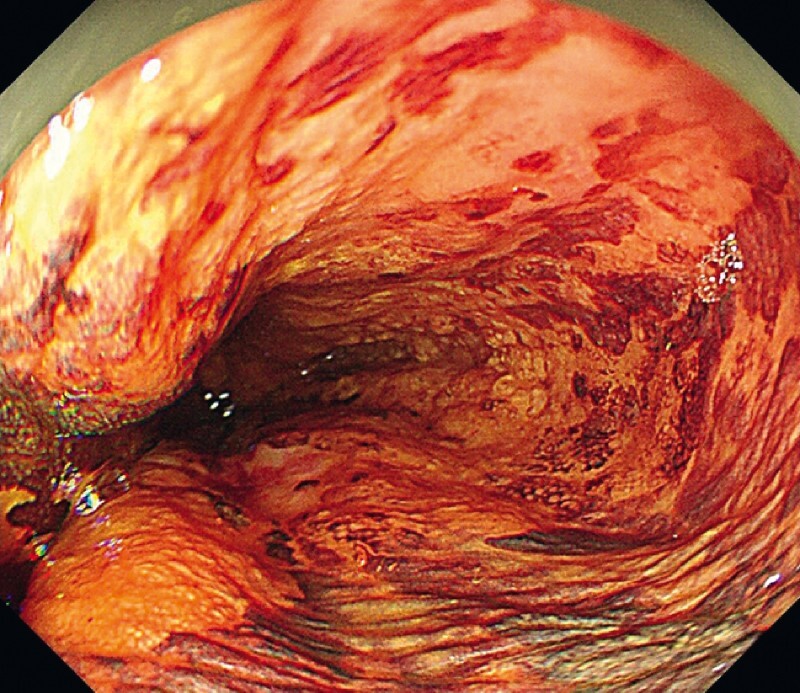
A circumferential esophageal lesion.

Repeated gas suction was applied intentionally every 15–20 minutes to reduce the gastric pressure. However, abrupt massive gastric bleeding with a large amount of blood gushing up into the esophagus was noted approximately 45 minutes after the operation started, when we were establishing a submucosal tunnel. The endoscope was immediately reinserted into the stomach for inspection. Multiple tears were observed, one of which was 3 cm in size and full thickness, with oozing extraluminal omental vessels ( Video 1 ). The oozing was stopped successfully by coagulating forceps. The ESTD procedure was then continued and completed uneventfully, with a total procedure time of 116 minutes. Finally, en bloc resection was achieved without any muscularis injury ( Fig. 2 ). After successfully managing the esophageal wound, we sutured the large perforation in the stomach using the endoloop string method and closed all other tears with clips ( Fig. 3 ).

**Video 1** Multiple gastric tears including a 3-cm perforation with oozing extraluminal omental vessels were observed during endoscopic submucosal tunnel dissection for an early circumferential esophageal cancer.


**Fig. 2:**
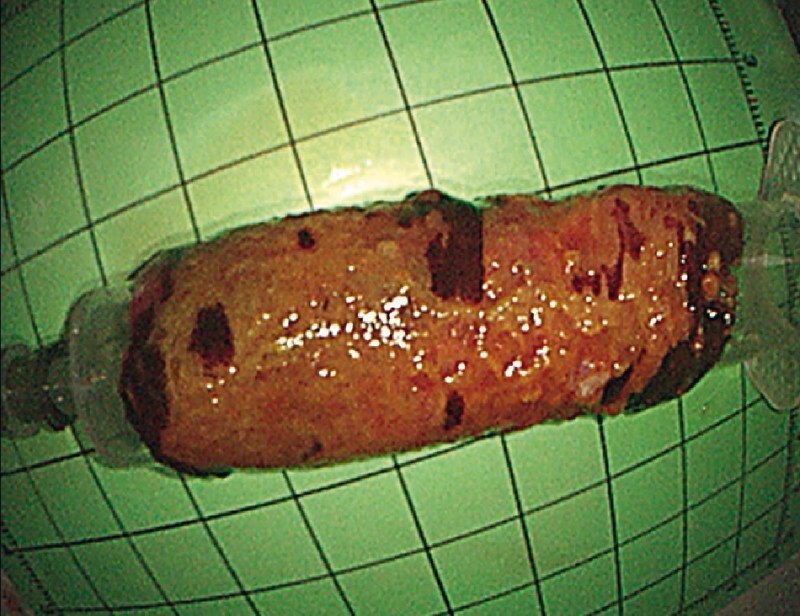
En bloc circumferentially resected specimen presented over a plastic tube.

**Fig. 3:**
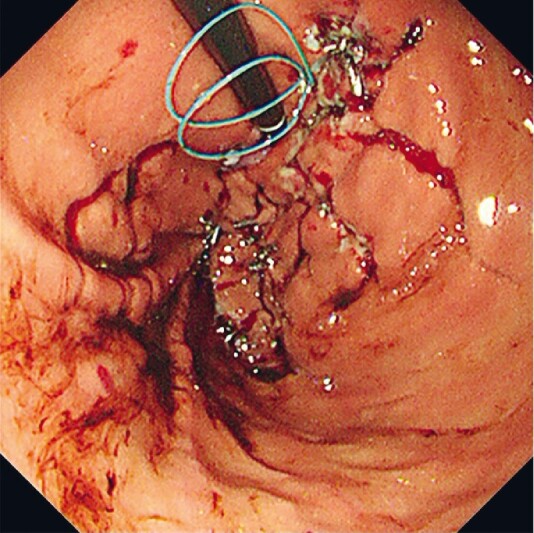
View of the stomach after closure of the perforation site and the multiple gastric tears.

The patient did well after the procedure and was discharged on postoperative Day 5 without any further adverse events. Follow-up endoscopy 3 months later showed good healing at all tearing sites ( Fig. 4 ).

**Fig. 4:**
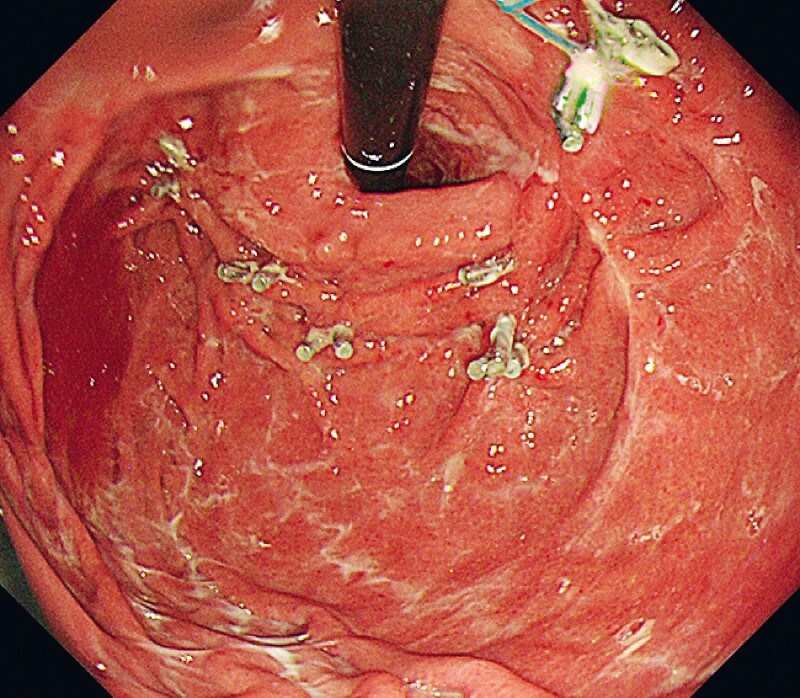
Follow-up endoscopy after 3 months.

Endoscopy_UCTN_Code_CPL_1AH_2AZ

## Notes

Endoscopy E-Videos

https://eref.thieme.de/e-videos
*E-Videos* is an open access online section of the journal *Endoscopy* , reporting on interesting cases and new techniques in gastroenterological endoscopy. All papers include a high-quality video and are published with a Creative Commons CC-BY license. Endoscopy E-Videos qualify for HINARI discounts and waivers and eligibility is automatically checked during the submission process. We grant 100% waivers to articles whose corresponding authors are based in Group A countries and 50% waivers to those who are based in Group B countries as classified by Research4Life (see: https://www.research4life.org/access/eligibility/ ). This section has its own submission website at https://mc.manuscriptcentral.com/e-videos

## References

[1] Chen W Zhu X N Wang J Risk factors for Mallory–Weiss tear during endoscopic submucosal dissection of superficial esophageal neoplasms World J Gastroenterol 2019 25 5174 5184 31558865 10.3748/wjg.v25.i34.5174 PMC6747285

[2] Lian J Chu Y Chen T Outcome of a novel self-control stricture-preventing water balloon for complete circular esophageal endoscopic submucosal dissection Surg Endosc 2023 37 290 297 35930071 10.1007/s00464-022-09456-8

